# ConMIL: interactive and contrastive text-guided multiple instance learning for whole slide image classification

**DOI:** 10.1093/bioinformatics/btag679

**Published:** 2026-09-15

**Authors:** Anxuan Han, Alexandra Jolley, Lisa M Butler, Weitong Chen

**Affiliations:** South Australian Immunogenomics Cancer Institute (SAiGENCI), Adelaide University, Adelaide, South Australia 5005, Australia; South Australian Immunogenomics Cancer Institute (SAiGENCI), Adelaide University, Adelaide, South Australia 5005, Australia; SA Pathology, Anatomical Pathology and Genetics and Molecular Pathology Directorates, Adelaide, South Australia 5000, Australia; South Australian Immunogenomics Cancer Institute (SAiGENCI), Adelaide University, Adelaide, South Australia 5005, Australia; Precision Cancer Medicine Theme, South Australian Health and Medical Research Institute (SAHMRI), Adelaide, South Australia 5000, Australia; School of Computer Science and Information Technology, Adelaide University, Adelaide, South Australia 5005, Australia

## Abstract

**Motivation:**

Whole-slide image (WSI) classification in computational pathology typically relies on Multiple Instance Learning (MIL) for weakly supervised analysis. Recent pathology vision-language models have inspired text-guided approaches, but these methods typically use text for representation alignment or region localization, rather than directly incorporating semantic signals into MIL attention weighting. Furthermore, these approaches often rely on static prompts and provide limited insight into the learned nonlinear transformations performed by the classifier.

**Results:**

We propose ConMIL, an interactive contrastive text-guided MIL framework for WSI classification. At its core, ConMIL introduces a contrastive semantic-guided attention mechanism that uses paired positive and negative pathology-specific text embeddings to directly modulate MIL attention weighting. This mechanism is complemented by human-in-the-loop prompt refinement to improve semantic specificity and a Kolmogorov–Arnold Network (KAN) classifier that enables visualization and quantitative inspection of learned nonlinear transformations. Experiments on CAMELYON16, TCGA-BRCA, and BRACS demonstrate that ConMIL consistently outperforms representative MIL baselines while producing pathology-consistent attention heatmaps and inspectable nonlinear transformations.

**Availability:**

The source code for ConMIL is available at https://github.com/anxuanhan/ConMIL

## 1 Introduction

Whole-slide images (WSIs) are high-resolution digital scans of hematoxylin and eosin (H&E)-stained histopathology slides and play a central role in computational pathology, enabling automated assessment of tissue morphology for cancer diagnosis, prognosis, and subtype classification (Lu *et al.* 2021, Nan *et al.* 2025). However, WSIs are gigapixel-scale images containing hundreds of thousands of patches, making dense manual annotation labor-intensive and clinically impractical (Srinidhi *et al.* 2021). In clinical practice, pathologists often need to examine extensive tissue regions to identify sparse metastatic lesions or subtle morphological abnormalities, making diagnosis time-consuming and prone to inter-observer variability. Multiple Instance Learning (MIL) has emerged as an effective tool to address these challenges, treating each slide as a bag of instances and enabling model training using only slide-level labels (Ilse *et al.* 2018, Li *et al.* 2021, Lu *et al.* 2021, Shao *et al.* 2021). By aggregating discriminative patch-level representations, MIL frameworks have demonstrated strong performance across a range of pathological tasks, including tumor detection, grading, and molecular subtype prediction (Yu *et al.* 2021, Javed *et al.* 2022, Li *et al.* 2023). Despite these advances, most existing MIL methods remain fundamentally image-driven and lack explicit incorporation of pathological semantic knowledge. As a result, attention weights are typically learned purely from image features, without leveraging the rich domain expertise accumulated by pathologists through decades of clinical practice.

Recent pathology vision-language models (VLMs), such as CONCH (Lu *et al.* 2024) and PLIP (Huang *et al.* 2023), align histopathological features with textual descriptions for knowledge-driven WSI analysis. Prompt-learning approaches such as CoOp (Zhou *et al.* 2022) and pathology-specific frameworks including FiVE (Li *et al.* 2024), PathTree (Li *et al.* 2026) and mTREE (Liu *et al.* 2025) incorporate pathological semantic information through representation alignment, region localization, or structured semantic guidance. More broadly, recent studies have integrated histology with spatial transcriptomics, extending computational pathology beyond image–text-based approaches (Chen *et al.* 2025, Zhang *et al.* 2025). However, the explicit use of paired positive and negative pathological semantics to directly modulate MIL attention remains underexplored.

In practice, the quality of pathological text prompts critically determines semantic retrieval specificity (Yang *et al.* 2025). Prompts generated by general-purpose large language models (LLMs) or manually designed fixed templates often become anchored to superficial visual cues, such as color, texture, or isolated cellular appearance, rather than pathology-relevant morphological and architectural features. As a result, the retrieved top-ranked patches may not always correspond to the intended pathological morphology described by the initial prompts. Human-in-the-loop review and iterative prompt refinement can therefore improve semantic reliability and retrieval specificity. Interactive machine learning paradigms have demonstrated that iterative expert feedback can substantially improve task-specific adaptation (Wu *et al.* 2022, Mosqueira-Rey *et al.* 2023). However, such interactive refinement mechanisms remain largely underexplored in text-guided WSI classification tasks.

Furthermore, interpretability in MIL is commonly explored through attention heatmaps, which visualize spatial regions emphasized during slide-level aggregation. However, such spatial visualization does not characterize the internal feature transformations performed by the classifier. Most MIL frameworks employ multilayer perceptrons (MLPs) for slide-level classification, in which nonlinear transformations are distributed across weighted connections and fixed activation functions (Lipton 2018). The recently proposed Kolmogorov–Arnold Network (KAN) provides an alternative by learning adaptive univariate functions on network edges (Liu *et al.* 2025). These learned edge functions can be directly visualized and quantitatively analyzed, enabling explicit inspection of nonlinear feature transformations within the classifier. KAN has demonstrated competitive performance across several deep learning tasks (Cheon 2024, Vaca-Rubio *et al.* 2024, Dutta *et al.* 2025). However, its application in computational pathology and WSI-based classification remains largely unexplored.

To address these limitations, we propose ConMIL, an interactive contrastive text-guided multiple instance learning framework for WSI classification. ConMIL employs a contrastive semantic-guided attention mechanism that incorporates semantic knowledge from paired positive and negative pathology-specific prompts into the learned patch-level attention weights, thereby highlighting diagnostically relevant regions while suppressing confounding regions. Building on this core design, ConMIL further incorporates human-in-the-loop prompt refinement to improve the pathological specificity of semantic guidance and a KAN classifier with learnable edge functions for direct inspection of nonlinear feature transformations. Extensive experiments on CAMELYON16, TCGA-BRCA, and BRACS demonstrate the effectiveness of the proposed framework across WSI classification tasks.

## 2 Methods

**Figure 1:**
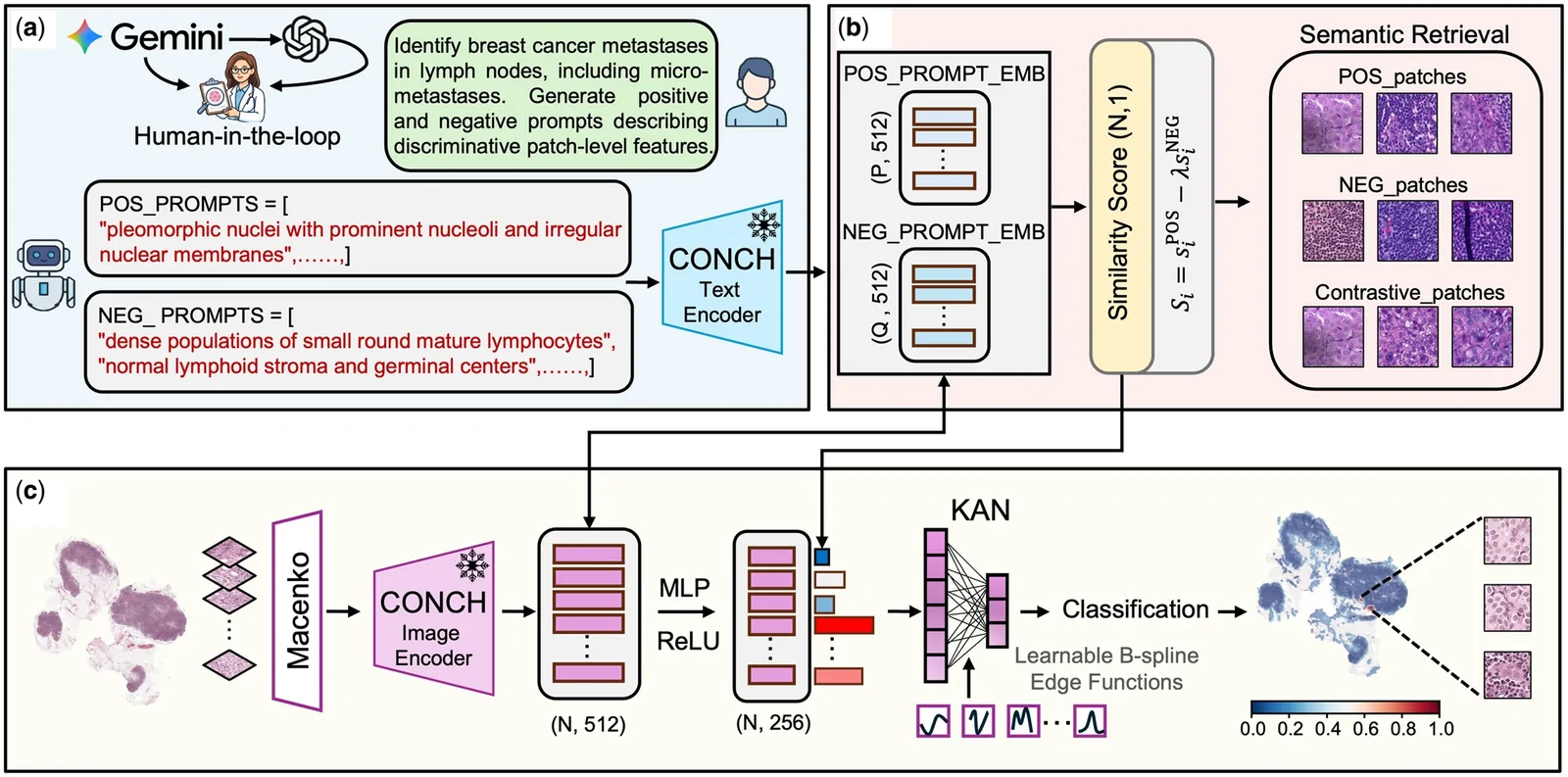
Overview of the ConMIL framework. (a) Human-in-the-loop construction and iterative refinement of positive and negative pathological text prompts, followed by semantic encoding using the CONCH text encoder. (b) Contrastive semantic retrieval module for patch retrieval using positive and negative prompt embeddings. (c) ConMIL classification pipeline. Whole-slide image patches are normalized, encoded by the CONCH image encoder, and aggregated through contrastive text-guided attention learning for slide-level classification using a KAN classifier with learnable B-spline edge functions. Representative attention heatmaps and top-ranked patches are shown.

We propose ConMIL, an interactive contrastive text-guided multiple instance learning framework for whole-slide image (WSI) classification that integrates pathological semantic knowledge into weakly supervised attention learning (Fig. 1). The framework consists of three stages. First (Fig. 1a), pathology-specific positive and negative text prompts are generated with the assistance of large language models and iteratively refined through pathologist-guided visual feedback to obtain pathology-consistent descriptions (see Section 2.3). The finalized prompts are subsequently encoded into semantic embeddings using the text encoder of the corresponding pathology VLM. Second (Fig. 1b), contrastive semantic similarity scores are computed between patch-level image features and prompt embeddings and subsequently incorporated into the MIL attention mechanism to guide attention towards pathology-relevant regions (see Section 2.4). Third (Fig. 1c), whole-slide image patches are encoded using the corresponding image encoder and aggregated through a text-guided multiple instance learning framework, where semantic similarity scores are incorporated as semantic attention biases prior to slide-level classification using a KAN classifier with learnable B-spline edge functions (see Sections 2.4–2.6).

### 2.1 Data collection and preparation

Three publicly available datasets were used to evaluate ConMIL. CAMELYON16 (Ehteshami Bejnordi *et al.* 2017) is a benchmark dataset for breast cancer lymph node metastasis detection. Following the official train–test split, 270 WSIs were used for training and 129 for independent testing, with 20% of the training set further reserved for validation using stratified sampling. TCGA-BRCA comprised 834 breast cancer WSIs from two major histological subtypes: invasive ductal carcinoma (IDC, *n* = 662) and invasive lobular carcinoma (ILC, *n* = 172). One representative WSI per patient was retained before partitioning; 10% of the data were reserved as an independent test set, and five-fold stratified cross-validation was performed on the remaining slides for training and validation. BRACS (Brancati *et al.* 2022) is a breast carcinoma subtyping dataset containing 547 WSIs grouped into benign (BT, *n* = 265), atypical (AT, *n* = 89), and malignant (MT, *n* = 193) categories. The training, validation, and test sets were defined according to the official BRACS split files.

### 2.2 WSI preprocessing and feature extraction

WSIs were preprocessed using a patch-based pipeline. For each slide, foreground tissue regions were identified using tissue masks, and patches were extracted at pyramid level 1, corresponding to approximately 20× magnification for CAMELYON16 and 10× magnification for TCGA-BRCA and BRACS. The higher magnification was used for CAMELYON16 to preserve cellular detail required for detecting small metastatic foci, whereas the lower magnification was selected for TCGA-BRCA and BRACS to capture broader tissue architecture relevant to histological subtype classification.

Patches containing more than 60% high-intensity pixels (grayscale intensity > 230) were discarded to exclude blank and adipose tissue regions. The remaining patches were resized to 224 × 224 pixels to match the input resolution of the image encoder. To reduce staining variability across slides, color normalization was performed using the Macenko method (Macenko *et al.* 2009) with a dataset-specific reference image for each cohort.

Patch-level features were primarily extracted using CONCH (ViT-B/16), a vision-language foundation model pretrained on pathology images (Lu *et al.* 2024). Each patch was encoded into a 512-dimensional L2-normalized feature vector. For robustness analysis, patch-level features were additionally extracted using PLIP under the same preprocessing pipeline. The extracted patch features were subsequently stored in HDF5 format for downstream multiple instance learning.

### 2.3 Text prompt construction

Candidate prompts were initially drafted using large language models (LLMs), including GPT-4 and Gemini (Achiam *et al.* 2023, Team *et al.* 2023), based on task-specific pathological descriptions, and were subsequently refined through iterative expert review. For CAMELYON16, prompts were designed to describe metastatic and normal tissue regions. For TCGA-BRCA, separate prompt sets were constructed for Invasive Lobular Carcinoma (ILC) and Invasive Ductal Carcinoma (IDC) to reflect their distinct histological morphology. During classification, subtype-specific prompt sets were treated as positive or negative relative to the target subtype. For BRACS, class-specific prompts were constructed for benign (BT), atypical (AT), and malignant (MT) categories, with the target class treated as positive and the remaining classes as negative semantic information.

Since LLM-generated descriptions may not fully align with the semantic space of pathology vision-language models, prompts were refined through pathologist-guided visual feedback. Prompts and image patches were encoded using the text and image encoders of the same pathology vision-language model, and cosine similarity was used to retrieve the top-ranked patches exclusively from the training set. A pathologist reviewed these patches and iteratively refined prompts based on morphologically inconsistent retrievals. Refinement stopped when the top 20 retrieved patches were all consistent with the intended class and pathological morphology. The resulting prompts were then frozen before validation and testing. To assess reproducibility, a second pathologist independently repeated the refinement procedure using the same initial prompts, training data, retrieval protocol, and stopping criterion on both CAMELYON16 and TCGA-BRCA. Both pathologists reached the predefined stopping criterion within two refinement rounds on both datasets. Prompts refined by the first pathologist were used for the main experiments, whereas those from the second pathologist were used for reproducibility assessment. The complete prompt refinement trajectories and final downstream performance are provided in Table S1.

The finalized prompts were encoded to obtain text features. For each patch with feature $x_{i}\in R^{512}$, the similarity score against the positive and negative prompt sets was computed as:


(1) sik=1|Tk|∑j=1|Tk|cos⁡(xi,tjk), k∈{POS,NEG}

where $\left|T^{k}\right|$ denotes the number of prompts in set k, and $t_{j}^{k}$ represents the text embedding of the j-th prompt. The contrastive similarity score for each patch was then computed as:


(2) si=siPOS-λsiNEG

where λ controls the influence of negative prompts and was fixed at 0.5 based on validation sensitivity analysis. The resulting contrastive $s_{i}$ provides a patch-level semantic signal reflecting the relative alignment of each patch with the target category and is subsequently incorporated into the attention mechanism described in Section 2.4.

### 2.4 Text-guided multiple instance learning

We formulate WSI classification as a MIL problem, where each slide is represented as a bag containing N instances with corresponding patch-level feature vectors ${\left\{x_{i}\right\}}_{i=1}^{N}$, where $x_{i}\in R^{512}$ is extracted from the image encoder and L2-normalized. Only slide-level labels are used for supervision.

#### 2.4.1 Feature projection

Each patch feature is first projected into a hidden representation using a linear transformation followed by ReLU activation and dropout regularization:


(3) hi=ReLU(Whxi+bh), hi∈R256

#### 2.4.2 Contrastive text-guided attention

To aggregate patch-level representations, we adopt a gated attention mechanism to compute attention scores for each patch:


(4) ai=Wa⊤(tanh⁡(Wvhi)⊙σ(Wuhi))

where $W_{v}$, $W_{u}$, and $W_{a}$ are learnable weight matrices, σ(⋅) denotes the sigmoid activation function, and ⊙ represents element-wise multiplication.

To incorporate pathological semantic guidance derived from the text prompts described in Section 2.3, the contrastive similarity score $s_{i}$ is incorporated into the attention score as an additive semantic bias:


(5) a∼i=ai+α⋅si

where α is a learnable scalar controlling the contribution of text guidance. The coefficient α was initialized to 0.5 in all experiments and optimized as an unconstrained learnable scalar without any sign or range constraints. Since both image and text embeddings were L2-normalized, the cosine-based semantic scores remained bounded before scaling by the learnable coefficient α. This formulation injects pathology-aware semantic priors into MIL attention logits as semantic attention biases. Patches with higher semantic similarity to positive pathological prompts receive increased attention scores, whereas semantically irrelevant or confounding patches are relatively suppressed through lower contrastive scores. The final attention weights are obtained using softmax normalization:


(6) πi=exp⁡(ai∼)∑j=1Nexp⁡(aj∼)

#### 2.4.3 Bag aggregation and classification

The bag-level representation is computed as the attention-weighted sum of patch embeddings:


(7) z=∑i=1Nπihi

The aggregated bag representation $z\in R^{256}$ is subsequently passed to a classification head for slide-level prediction. In the standard variant [ConMIL (MLP)], the classifier consists of a two-layer multilayer perceptron (MLP). In the KAN variant (ConMIL), the MLP is replaced by a Kolmogorov–Arnold Network (KAN) with learnable B-spline edge functions (see Section 2.5).

#### 2.4.4 Multi-class extension

For multi-class classification, the contrastive semantic scoring and attention mechanism were extended in a class-specific manner, with each target class treated as positive semantic information and the remaining classes as negative semantic information. The detailed formulation is provided in Supplementary Data.

### 2.5 KAN-based classification

In the KAN-based ConMIL, the MLP classification head is replaced by a Kolmogorov–Arnold Network (KAN) (Liu *et al.* 2025). The KAN classifier consists of two layers, mapping the 256-dimensional bag representation to a 128-dimensional hidden space followed by the output layer. Given the aggregated bag representation $z\in R^{256}$, the KAN classifier maps z to the final prediction through a composition of learnable edge functions φ(⋅). For quantitative analysis, the activation amplitude was defined as the difference between the maximum and minimum values of each learned edge function, and linearity was quantified using the R2 of a linear fit. Tumor–Normal differential amplitude was defined as the difference between the corresponding activation amplitudes for tumor and normal samples.

### 2.6 Implementation details

#### 2.6.1 Training

The model was trained using cross-entropy loss with slide-level labels. For each experiment, the checkpoint achieving the highest validation accuracy was selected for test-set evaluation. A random seed of 42 was used for all experiments.

#### 2.6.2 Baseline implementation

CoOp and FiVE were evaluated using the same CONCH-derived patch features and dataset partitions as ConMIL. CoOp was adapted using learnable class-specific prompts with class-wise Top-K pooling, while FiVE retained its visual-semantic interaction framework with class-specific pathology prompts replacing report-based textual inputs.

## 3 Results

### 3.1 Contrastive prompts improve pathology-relevant patch retrieval

To evaluate whether pathological text prompts can guide semantically relevant patch retrieval, we visualized patch retrieval results based on contrastive similarity scoring on CAMELYON16 and TCGA-BRCA. For CAMELYON16, positive prompts described morphological features of metastatic tumor regions, while negative prompts corresponded to non-target or confounding regions such as lymphocyte-rich areas and normal stromal tissue. For TCGA-BRCA subtype classification, ILC-specific and IDC-specific prompts were used as reciprocal positive and negative semantic descriptors for each subtype.

**Figure 2:**
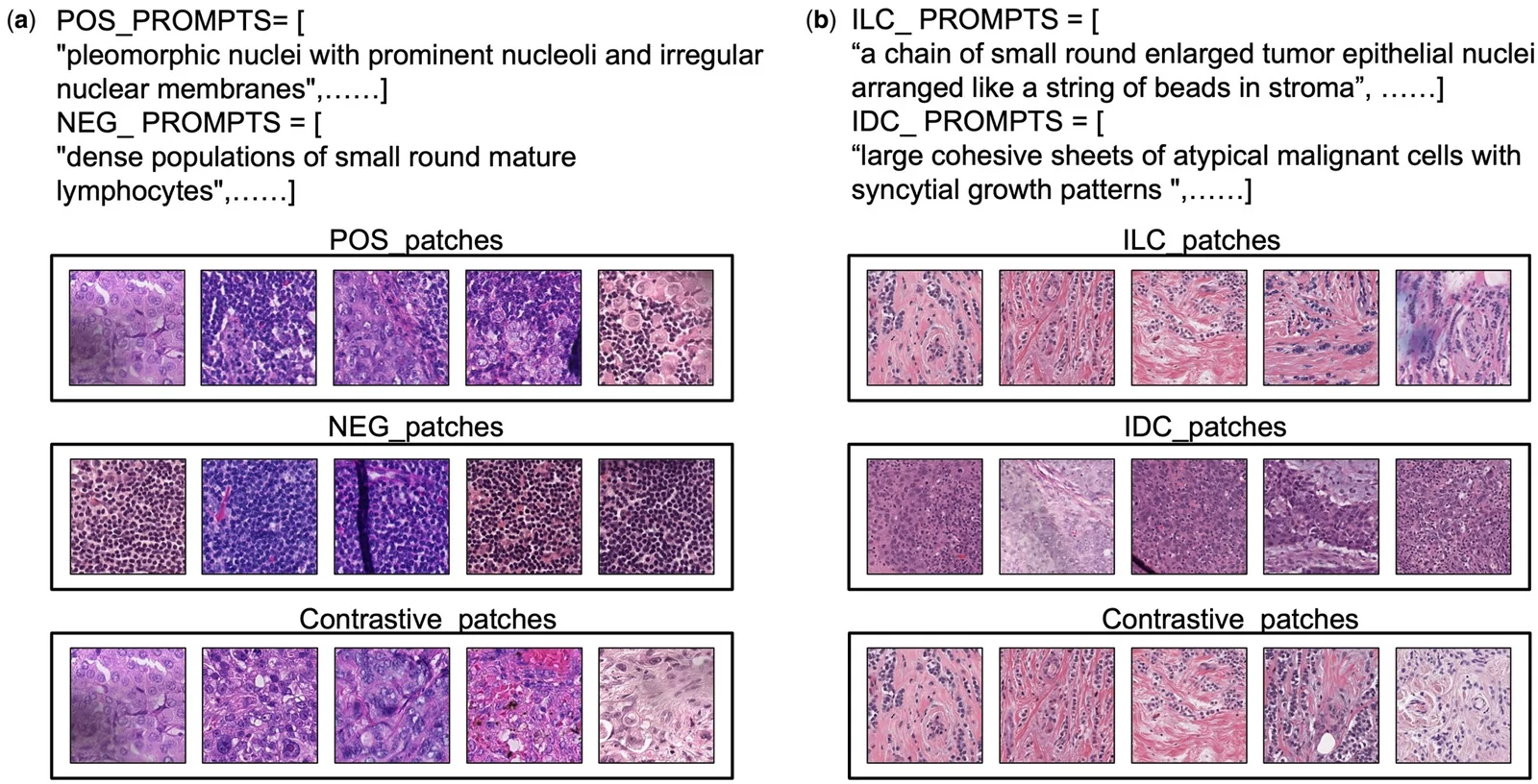
Contrastive semantic retrieval using pathological text prompts. (a) Retrieved CAMELYON16 patches using positive prompts alone, negative prompts alone, and contrastive semantic scoring for breast cancer metastasis detection. (b) Retrieved TCGA-BRCA patches using subtype-specific ILC and IDC prompts together with contrastive semantic scoring for subtype classification.

As shown in Fig. 2a, positive prompts on CAMELYON16 retrieved metastatic tumor regions but frequently retained lymphocyte-rich confounding areas, whereas contrastive scoring produced patches with higher tumor-specific morphological purity. In TCGA-BRCA subtype classification (Fig. 2b), reciprocal ILC and IDC prompts retrieved subtype-associated morphological patterns, and contrastive scoring further improved the specificity of subtype-related patch retrieval. Together, these results demonstrate that contrastive text-guided semantic scoring effectively discriminates pathology-relevant patches from confounding regions, providing more discriminative semantic signals for subsequent weakly supervised attention learning.

### 3.2 Human-in-the-loop semantic refinement improves pathology specificity

**Figure 3:**
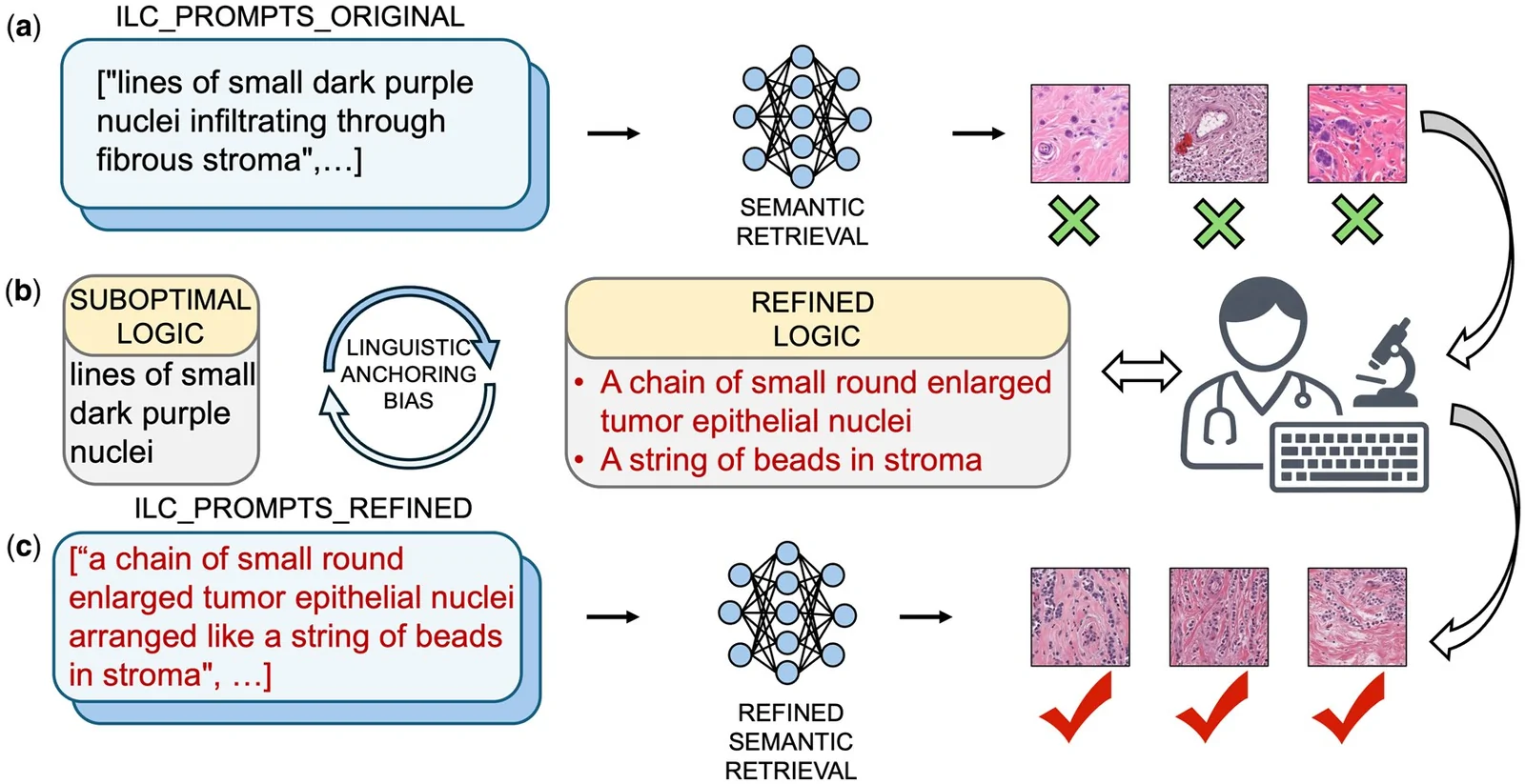
Human-in-the-loop semantic refinement of pathological text prompts. (a) Initial ILC prompts and corresponding retrieved patches. (b) Expert-guided refinement replaces the initial prompts with pathology-grounded morphological concepts through iterative pathologist feedback. (c) Refined ILC prompts and corresponding retrieved patches.

While contrastive semantic scoring effectively retrieves pathology-relevant patches given well-designed prompts, retrieval quality remained sensitive to the semantic specificity of the text descriptions. To illustrate the interactive semantic refinement mechanism, we present ILC subtype classification on TCGA-BRCA as a representative example. Initial prompts generated by large language models primarily relied on low-level visual appearance cues, such as “lines of small dark purple nuclei infiltrating through fibrous stroma,” emphasizing isolated cellular features rather than the tissue-level architectural patterns used in pathological diagnosis. As a result, the retrieved patches frequently corresponded to morphologically irrelevant regions (Fig. 3a), suggesting that such descriptions may introduce a linguistic anchoring bias that fails to capture pathology-relevant structural concepts and growth patterns.

These retrieval failures were subsequently reviewed by a pathologist, who refined the prompts to emphasize pathology-grounded architectural and structural features rather than superficial visual appearance. For example, “lines of small dark purple nuclei” was refined into “a chain of small round enlarged tumor epithelial nuclei arranged like a string of beads in stroma,” shifting the description from isolated cellular appearance toward the characteristic single-file infiltration pattern of ILC (Fig. 3b). As shown in Fig. 3c, the refined prompts produced more pathology-specific retrieval patterns and consistently identified ILC-associated morphological regions missed by the original prompts. Quantitative evaluation showed that prompt refinement improved or maintained downstream performance relative to the initial LLM-generated prompts. The complete refinement trajectories for two pathologists on CAMELYON16 and TCGA-BRCA, together with their comparable downstream performance, are provided in Table S1.

### 3.3 ConMIL improves WSI classification performance

To comprehensively evaluate ConMIL, we compared the full ConMIL framework against four representative MIL baselines, including DSMIL, ABMIL, CLAM, and TransMIL (Ilse *et al.* 2018, Li *et al.* 2021, Lu *et al.* 2021, Shao *et al.* 2021), as well as two text-guided baselines, CoOp (Zhou *et al.* 2022) and FiVE (Li *et al.* 2024), across CAMELYON16, TCGA-BRCA, and BRACS. As shown in Table 1, ConMIL consistently achieved the best overall performance across all three datasets using CONCH-derived features, reaching AUCs of 0.9865, 0.9505, and 0.8874 on CAMELYON16, TCGA-BRCA, and BRACS, respectively. ConMIL also achieved the highest ACC and F1/Macro-F1 scores across the three datasets, supporting its effectiveness in both binary and multi-class WSI classification. To assess robustness to different pathology VLM encoders, we repeated the experiments using PLIP-derived features on CAMELYON16 and TCGA-BRCA. ConMIL achieved the best overall performance among the PLIP-based models, although absolute performance was lower than with CONCH (Table S2). Unless otherwise specified, all subsequent analyses were conducted using CONCH-derived features.

**Table 1:** Comparison of ConMIL with representative MIL and text-guided baselines using CONCH-derived features across CAMELYON16, TCGA-BRCA, and BRACS.

Model	CAMELYON16			TCGA-BRCA			BRACS		
	AUC	ACC	F1	AUC	ACC	F1	AUC	ACC	Macro-F1
DSMIL	0.9786	0.9457	0.9278	0.9475 ± 0.0063	0.9238 ± 0.0095	0.9523 ± 0.0062	0.8147	0.5862	0.5592
ABMIL	0.9689	0.9225	0.8889	0.9495 ± 0.0210	0.9187 ± 0.0148	0.9494 ± 0.0094	0.8839	0.7471	0.7425
CLAM	0.9551	0.9225	0.8936	0.9487 ± 0.0065	0.9286 ± 0.0199	0.9563 ± 0.0113	0.8824	0.7356	0.7329
TransMIL	0.9852	0.9612	0.9580	0.9335 ± 0.0051	0.9238 ± 0.0095	0.8738 ± 0.0213	0.8799	0.6667	0.6519
CoOp	0.9819	0.9612	0.9362	0.9266 ± 0.0068	0.9241 ± 0.0058	0.9511 ± 0.0037	0.8745	0.6552	0.6341
FiVE	0.9756	0.9612	0.9324	0.9495 ± 0.0037	0.9342 ± 0.0033	0.9541 ± 0.0137	0.8723	0.7126	0.6914
**ConMIL**	**0.9865**	**0.9690**	**0.9583**	**0.9505 ± 0.0076**	**0.9429 ± 0.0048**	**0.9647 ± 0.0030**	**0.8874**	**0.7586**	**0.7507**

Bold values indicate the best performance.

Ablation studies were conducted to evaluate the contributions of semantic guidance, prompt refinement, and the KAN classifier. As shown in Table 2, both positive-only and negative-only guidance generally improved over the baseline model without any text-based semantic guidance, whereas unrelated and swapped prompts performed worse, supporting the importance of pathology-relevant semantic guidance.

**Table 2:** Component-wise ablation study of ConMIL using CONCH-derived features on CAMELYON16 and TCGA-BRCA.

Ablation configuration						CAMELYON16			TCGA-BRCA		
Stage	Setting	Prompt source	Positive prompt	Negative prompt	Classifier	AUC	ACC	F1	AUC	ACC	F1
Baseline	Baseline	–	–	–	MLP	0.9763	0.9147	0.8764	0.9422 ± 0.0115	0.9357 ± 0.0048	0.9494 ± 0.0024
	Baseline	–	–	–	KAN	0.9735	0.9380	0.9167	0.9498 ± 0.0056	0.9310 ± 0.0089	0.9571 ± 0.0054
Prompt ablation	Positive-only	Pathologist	✓	–	MLP	0.9770	0.9457	0.9278	0.9424 ± 0.0097	0.9381 ± 0.0190	0.9609 ± 0.0123
	Negative-only	Pathologist	–	✓	MLP	0.9778	0.9380	0.9200	0.9424 ± 0.0038	0.9286 ± 0.0106	0.9561 ± 0.0059
	Swapped	Pathologist	Negative	Positive	MLP	0.9696	0.9070	0.8867	0.9414 ± 0.0093	0.9281 ± 0.0117	0.9238 ± 0.0065
	Unrelated	Unrelated	Unrelated	Unrelated	MLP	0.9658	0.8759	0.8949	0.9398 ± 0.0086	0.9333 ± 0.0095	0.9490 ± 0.0055
	LLM-generated	LLM	✓	✓	MLP	0.9799	0.9535	0.9388	0.9428 ± 0.0097	0.9385 ± 0.0090	0.9619 ± 0.0097
	Refined	Pathologist	✓	✓	MLP	0.9849	0.9535	0.9388	0.9480 ± 0.0124	0.9427 ± 0.0089	0.9639 ± 0.0058
Classifier ablation	Refined (Full model)	Pathologist	✓	✓	KAN	**0.9865**	**0.9690**	**0.9583**	**0.9505 ± 0.0076**	**0.9429 ± 0.0048**	**0.9647 ± 0.0030**

LLM-generated prompts represent the initial prompts before human refinement. Bold values indicate the best performance.

Among the MLP-based settings, human-refined contrastive prompts achieved the highest AUCs, reaching 0.9849 and 0.9480 on CAMELYON16 and TCGA-BRCA, respectively. KAN alone did not consistently outperform the MLP baseline, but under the same refined contrastive guidance, KAN achieved the best overall performance. However, the substantially larger parameter count of KAN prevents architecture-specific attribution of this difference.

We further examined the coefficients controlling semantic guidance. The learned α values obtained from the validation-selected checkpoints were 0.5383 on CAMELYON16 and 0.4937 ± 0.0293 on TCGA-BRCA (Table S4). Sensitivity analysis on the validation sets further showed that λ = 0.5 achieved the highest ACC among the evaluated settings (Supplementary Fig. S1).

### 3.4 ConMIL learns pathology-consistent attention patterns

**Figure 4:**
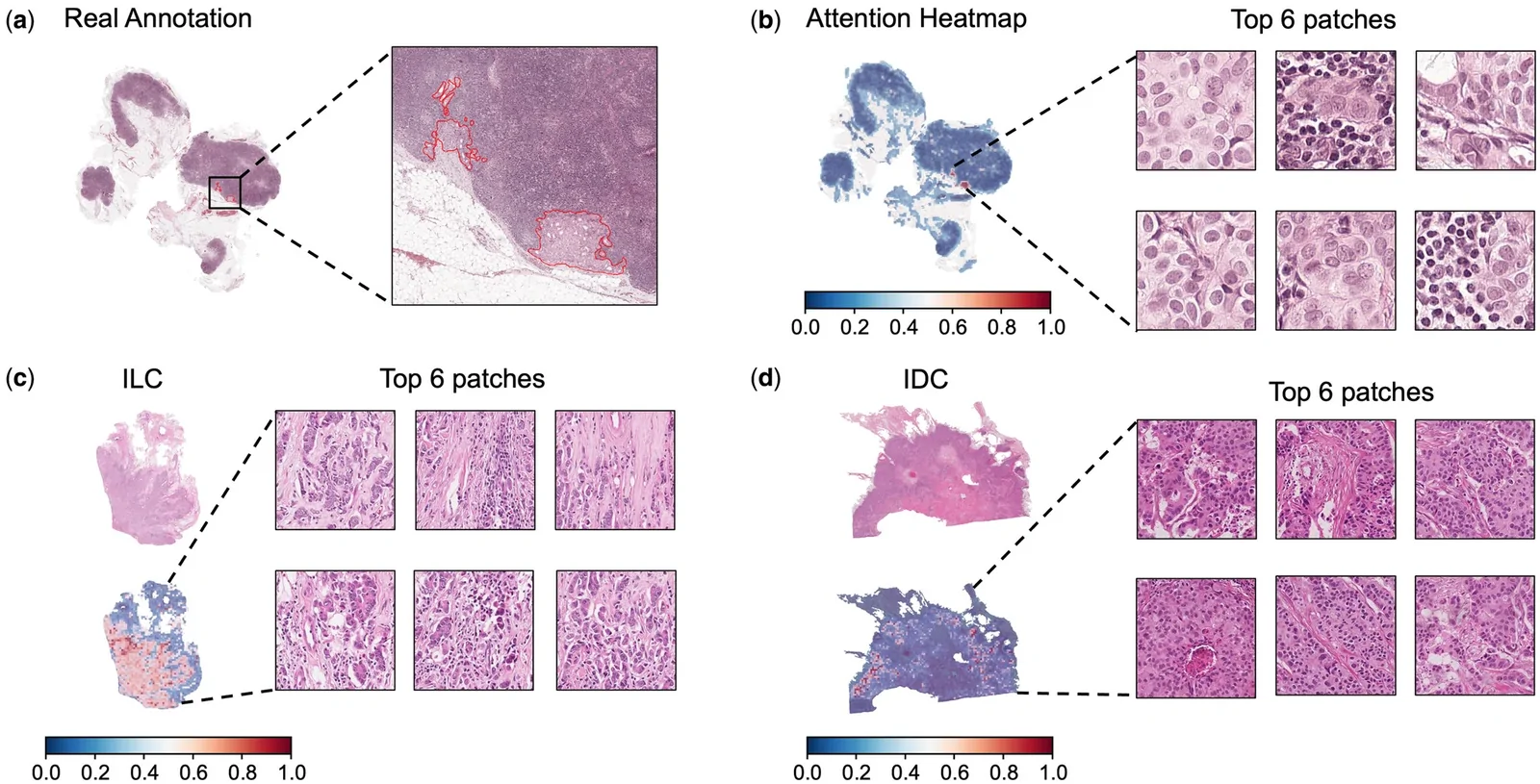
Visualization of pathology-consistent attention patterns generated by ConMIL. (a) Ground-truth annotation of metastatic regions in a CAMELYON16 whole-slide image. (b) Attention heatmap generated by ConMIL and corresponding top-ranked metastatic patches in CAMELYON16. (c) Attention heatmap and top-ranked patches for ILC in TCGA-BRCA. (d) Attention heatmap and top-ranked patches for IDC in TCGA-BRCA.

Beyond quantitative performance, we further examined whether ConMIL generates pathology-consistent attention patterns by visualizing attention heatmaps and top-ranked patches on CAMELYON16 and TCGA-BRCA. As shown in Fig. 4a and b, attention heatmaps highlighted tissue regions associated with metastatic tumor morphology. The associated top-ranked patches exhibited characteristic features of metastatic carcinoma, including pleomorphic nuclei, prominent nucleoli, and irregular nuclear contours.

For TCGA-BRCA subtype classification, ConMIL produced subtype-specific attention patterns consistent with known histological characteristics of ILC and IDC (Fig. 4c and d). In ILC slides, high-attention regions and top-ranked patches captured discohesive tumor cells and infiltrative growth patterns within the stroma. In contrast, IDC attention was concentrated on cohesive tumor regions composed of atypical malignant epithelial cells.

Together, these results suggest that ConMIL learns pathology-consistent attention patterns without requiring pixel-level supervision, indicating that pathological semantic knowledge embedded in text prompts effectively shapes attention learning toward diagnostically relevant tissue regions.

### 3.5 KAN enables inspection of learned nonlinear transformations

**Figure 5:**
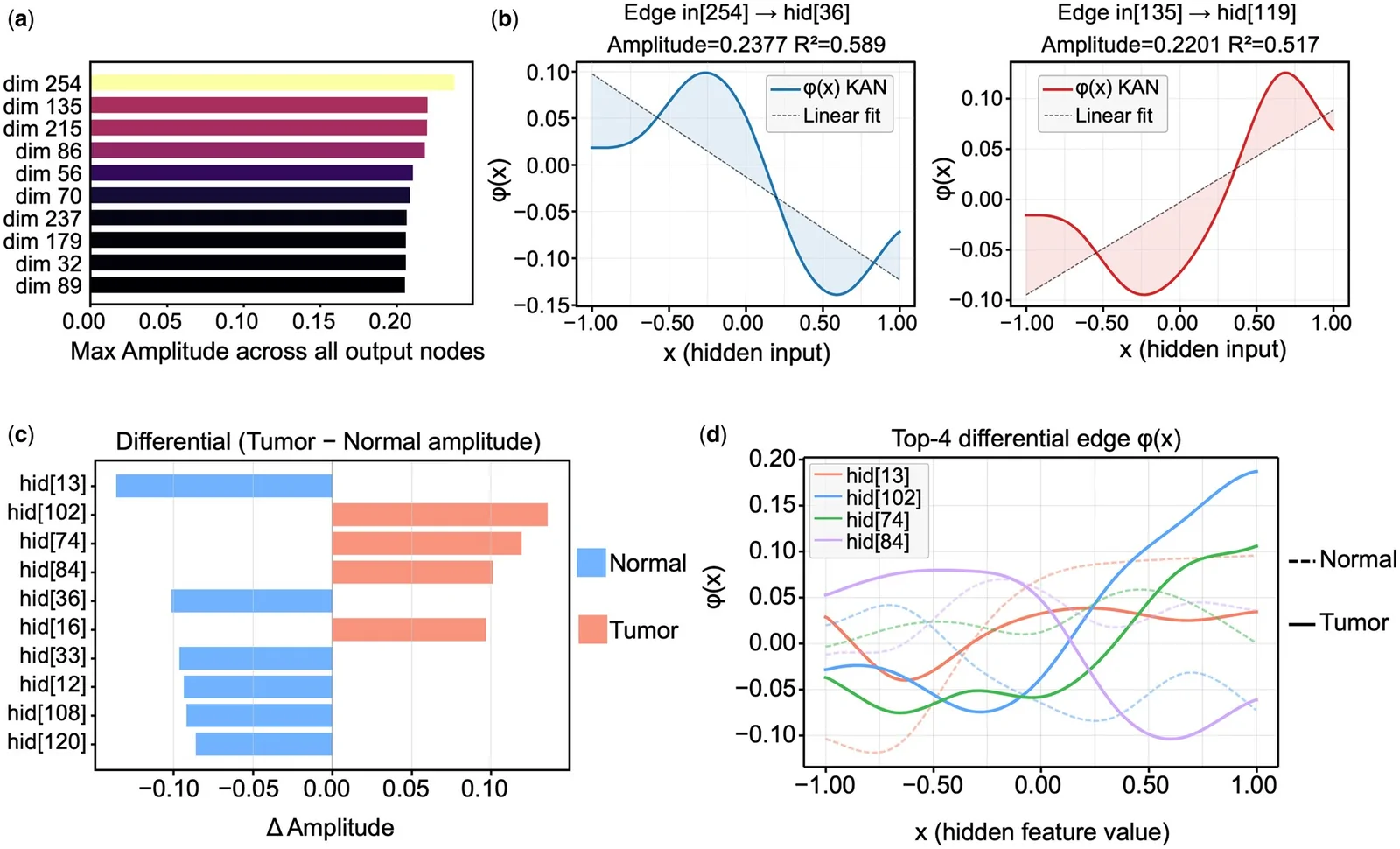
Inspection of learned nonlinear transformations in the KAN classifier. (a) Top-ranked feature dimensions with the largest activation amplitudes across KAN output nodes. (b) Representative nonlinear edge activation functions learned by the KAN classifier compared with linear fits. (c) Differential activation amplitudes between tumor and normal samples across hidden nodes. (d) Representative edge activation functions for tumor and normal states. Solid lines indicate tumor-associated activations, whereas dashed lines indicate normal-associated activations.

While attention heatmaps reveal where the model focuses, KAN provides complementary functional insights through its learnable edge-level activation functions. As shown in Fig. 5a, ranking input dimensions by maximum activation amplitude across all output nodes in the input-to-hidden layer identified dim 254 and dim 135 as two high-amplitude dimensions for further edge activation analysis. As shown in Fig. 5b, the corresponding edge functions exhibited clear nonlinear response patterns that deviated markedly from linear fits, with amplitudes of 0.2377 (*R*^2^ = 0.589) and 0.2201 (*R*^2^ = 0.517), respectively. These observations suggest that KAN exposes feature-specific nonlinear activation dynamics that are less directly inspectable in conventional MLP classifiers.

Differential amplitude analysis in the hidden-to-output layer further revealed class-specific activation patterns (Fig. 5c). Hidden nodes hid[102], hid[74], and hid[84] exhibited higher amplitudes in tumor samples, whereas hid[13] and hid[36] showed stronger activation in normal samples. Consistently, the corresponding edge activation curves displayed distinct response trajectories between tumor and normal groups, illustrating differential activation dynamics learned by the KAN classifier.

## 4 Discussion

This study demonstrates that explicitly incorporating contrastive semantic guidance into learned MIL attention can improve weakly supervised WSI classification. A central finding is that complementary positive and negative pathology-specific prompts provide more discriminative semantic guidance than positive prompts alone. Positive prompts encourage attention towards target-associated morphology, whereas negative prompts suppress semantically confounding regions, thereby improving attention separability under weak supervision. These findings suggest that contrastive semantic guidance facilitates discrimination between target and confounding tissue patterns under weak supervision.

A second key finding concerns the role of prompt quality in semantic retrieval specificity. Initial prompts generated by LLMs frequently anchored to low-level visual features susceptible to staining variability and lacking pathological grounding. The human-in-the-loop refinement experiments showed that descriptions emphasizing tissue architecture and pathological structure retrieved more pathology-relevant patches than descriptions focused on low-level visual appearance cues. This finding highlights linguistic anchoring bias as a practical challenge in text-guided computational pathology. Interactive prompt refinement therefore serves as a complementary component that improves the specificity of semantic guidance.

Several limitations should be acknowledged. First, the interactive refinement process currently relies on direct pathologist involvement, which may limit scalability in large-scale applications. Although LLM-assisted prompt generation may reduce the burden of manual refinement within the predefined stopping-criterion procedure, its reliability and pathological precision require further validation. Second, ConMIL may be less applicable to pathological entities that are difficult to describe using natural language, and validation on larger multi-institutional cohorts is needed to establish broader generalizability. Finally, although the learnable edge functions of KAN enable visualization and quantitative inspection of nonlinear transformations, their latent dimensions cannot yet be directly associated with specific pathological or biological concepts. Establishing such concept-level correspondence represents an important direction for future work. Nevertheless, ConMIL demonstrates that pathology-specific semantic knowledge can serve as an expert-refinable inductive bias for weakly supervised attention learning, providing a promising direction for more transparent and pathology-aware WSI analysis.

## Supplementary Material

btag679_Supplementary_Data

## Data Availability

The datasets used in this study are publicly available from the GDC Data Portal (https://portal.gdc.cancer.gov/), CAMELYON16 (https://camelyon16.grand-challenge.org/Download/), and BRACS (https://www.bracs.icar.cnr.it/).
