# Supplementary material for: ConMIL: interactive and contrastive text-guided multiple instance learning for whole slide image classification
Source: Bioinformatics. 2026 Sep 15;42(9):btag679. doi: 10.1093/bioinformatics/btag679 (PMC13614712; doi:10.1093/bioinformatics/btag679)
Supplement: Supplementary file 1 [file btag679_supplementary_data.pdf]

Supplementary Methods

Multi-class extension.

For multi-class classification with C classes, a class-specific prompt set T^c was constructed for each class c. For patch i, the semantic similarity to class c was computed as

$$r_i^c = \frac{1}{|T^c|} \sum_{j=1}^{|T^c|} \cos(\mathbf{x}_i, \mathbf{t}_j^c) \tag{S1}$$

where $\mathbf{x}_i$ denotes the patch embedding and $\mathbf{t}_j^c$ denotes the embedding of the j-th prompt for class c. For each candidate class, its class-specific prompt set was treated as positive semantic information, whereas the remaining $C-1$ classes were treated as negative semantic information. The class-specific contrastive score was therefore defined as

$$s_i^c = r_i^c - \frac{\lambda}{C-1} \sum_{k \neq c} r_i^k \tag{S2}$$

where λ controls the contribution of the remaining classes. This formulation measures the relative semantic alignment of each patch with candidate class c against the remaining classes and reduces to the binary contrastive formulation when $C = 2$.

The resulting class-specific semantic score was incorporated into the MIL attention logits as:

$$\tilde{a}_i^c = a_i + \alpha s_i^c \tag{S3}$$

where a_i is the image-derived gated attention logit and α is the learnable semantic guidance coefficient. Class-specific attention weights were then obtained by normalization across patches:

$$\pi_i^c = \frac{\exp\left((\tilde{a_i^c})\right)}{\sum_{j=1}^{N} \exp\left((\tilde{a_j^c})\right)} \tag{S4}$$

For each class, a class-specific bag representation was computed as:

$$\boldsymbol{z}^c = \sum_{i=1}^{N} \pi_i^c \, \boldsymbol{h}_i \tag{S5}$$

Each class-specific bag representation $\boldsymbol{z}^c$ was passed through the shared slide-level classifier f_θ, and the output corresponding to candidate class c was used as its class logit:

$$\ell_c = [f_\theta(\mathbf{z}^c)]_c \tag{S6}$$

where f_θ, denotes the shared classifier and $[\cdot]_c$ selects the output for class c. The resulting class logits were normalized using softmax.

At inference, the above procedure was performed for all C candidate classes without using ground-truth class information.

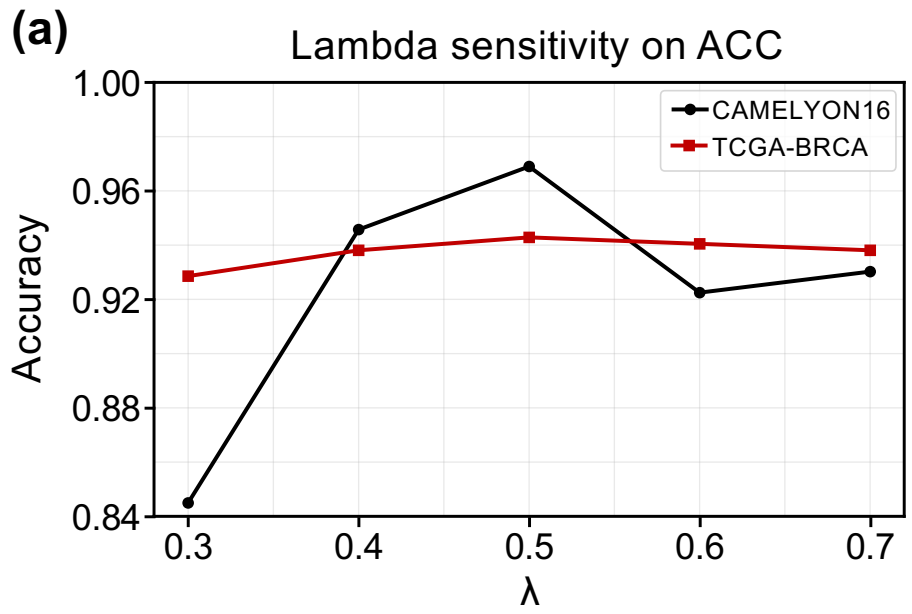

Supplementary Fig. S1. Sensitivity analysis of the negative-prompt weighting coefficient. λ values from 0.3 to 0.7 were evaluated on the validation sets of CAMELYON16 and TCGA-BRCA.

Alt text: Line plots showing the effect of the negative-prompt weighting coefficient λ on classification accuracy for CAMELYON16 and TCGA-BRCA validation sets, with the best overall performance observed at λ = 0.5.

Supplementary Table S1. Prompt refinement trajectories.

Dataset	Prompt Set	Prompt Type	Prompt
Camelyon16	Initial	Positive	breast cancer cells in lymph node
			tumor cells in lymph node tissue
			metastatic cancer cells
			abnormal cells in lymph node
	Initial	Negative	normal lymph node
			normal lymphoid tissue
			benign lymph node tissue
			normal cells in lymph node

Pathologist	Refinement Round	Prompt Type	Prompt	AUC	ACC	F1
Pathologist1	1	Positive	metastatic breast cancer cells infiltrating lymph node tissue			
			clusters of malignant epithelial cells in lymphoid tissue			
			atypical epithelial cells with enlarged irregular nuclei			
			metastatic tumor cell clusters within lymph node			
		Negative	lymph node tissue containing small mature lymphocytes			
			benign lymphoid tissue without malignant epithelial cells			
			normal lymphoid follicles and lymphocytes			
			reactive lymphoid tissue without metastatic tumor cells			
	2	Positive	pleomorphic nuclei with prominent nucleoli and irregular nuclear membranes	0.9865	0.969	0.9583
			tightly packed malignant epithelial cell clusters			
			metastatic adenocarcinoma in lymph node sinuses			
			high nuclear-to-cytoplasmic ratio in malignant cells			
		Negative	dense populations of small round mature lymphocytes			
			normal lymphoid stroma and germinal centers			
Pathologist2	1	Positive	metastatic epithelial cells infiltrating lymphoid tissue			
			cohesive clusters of atypical epithelial cells within lymph nodes			
			enlarged hyperchromatic nuclei with marked nuclear atypia			
			small metastatic tumor deposits surrounded by lymphocytes			
		Negative	densely packed small lymphocytes without atypical epithelial cells			
			preserved lymph node architecture with reactive lymphoid follicles			
			germinal centers surrounded by mature lymphocytes			
			benign lymphoid tissue without evidence of metastatic carcinoma			
	2	Positive	cohesive nests of malignant epithelial cells	0.9819	0.9612	0.9474
			clusters of pleomorphic carcinoma cells with abundant cytoplasm			
			large atypical epithelial cells with irregular nuclei and prominent nucleoli			
			malignant epithelial cell clusters with high nuclear-to-cytoplasmic ratio			
		Negative	densely packed small lymphocytes without atypical epithelial cells			
			preserved lymph node architecture with reactive lymphoid follicles			
			germinal centers surrounded by mature lymphocytes			
			benign lymphoid tissue without evidence of metastatic carcinoma			

Dataset	Prompt Set	Prompt Type	Prompt
TCGA-BRCA	Initial	ILC	lines of small dark purple nuclei infiltrating through fibrous stroma
			interrupted linear chains of dyscohesive small tumor cells
			individual small ovoid cells scattered in a targetoid or linear pattern
			sparse population of small monomorphic nuclei squeezed into thin rows
	Initial	IDC	cohesive clusters of invasive carcinoma cells
			irregular nests of malignant epithelial cells
			crowded tumor cells forming solid sheets
			atypical epithelial cells arranged in gland-like structures

Pathologist	Refinement Round	Prompt Type	Prompt	AUC	ACC	F1
Pathologist1	1	ILC	small dyscohesive tumor cells arranged in linear chains within stroma			
			individual small tumor cells infiltrating through fibrous stroma			
			narrow cords of relatively uniform tumor cells in a single-file pattern			
			linear strands of non-cohesive epithelial cells between stromal fibers			
		IDC	cohesive sheets of atypical malignant epithelial cells			
			solid nests of pleomorphic carcinoma cells infiltrating stroma			
			dense clusters of malignant epithelial cells with limited gland formation			
			enlarged atypical nuclei within cohesive tumor cell aggregates			
	2	ILC	a chain of small round enlarged tumor epithelial nuclei arranged like a string of beads in stroma	0.9505 ± 0.0076	0.9429 ± 0.0048	0.9647 ± 0.0030
			individual tumor cells infiltrating in a single-file linear row			
			slender cords of small uniform tumor cells permeating through collagenous stroma in single file			
			discontinuous linear strands of dyscohesive small epithelial cells			
		IDC	large cohesive sheets of atypical malignant cells with syncytial growth patterns			
			solid nest-like infiltration of enlarged pleomorphic tumor cells filling the stroma			
			broad expansive areas of dense malignant epithelium with no glandular lumens			
			pleomorphic enlarged nuclei embedded within tightly packed cohesive tumor clusters			
Pathologist2	1	ILC	dyscohesive small tumor cells infiltrating individually through fibrous stroma			
			single-file infiltration of uniform malignant cells between collagen fibers			
			loosely dispersed monomorphic carcinoma cells lacking cohesive architecture			
			targetoid arrangement of small tumor cells around ducts and stromal structures			
		IDC	cohesive nests of malignant epithelial cells invading desmoplastic stroma			
			irregular clusters of pleomorphic carcinoma cells with marked nuclear atypia			
			solid cohesive tumor aggregates composed of enlarged malignant epithelial cells			
			invasive carcinoma forming irregular cords and poorly formed tubular structures			
	2	ILC	single-file rows of small dyscohesive tumor cells infiltrating fibrous stroma	0.9463 ± 0.0047	0.9405 ± 0.0151	0.9625 ± 0.0099
			discontinuous linear chains of small monomorphic epithelial cells			
			isolated non-cohesive tumor cells dispersed individually through collagenous stroma			
			small uniform tumor cells arranged in targetoid patterns around ducts			
		IDC	cohesive clusters of malignant epithelial cells forming broad solid nests			
			densely packed carcinoma cells arranged in compact epithelial sheets			
			malignant epithelial cells forming irregular glandular and duct-like structures			
			large cohesive tumor aggregates composed of pleomorphic epithelial cells			

Supplementary Table S2. Comparison of ConMIL with representative MIL and text-guided baselines using PLIP-derived features across CAMELYON16 and TCGA-BRCA.

	CAMELYON16			TCGA-BRCA		
Model	AUC	ACC	F1	AUC	ACC	F1
DSMIL	0.8648	0.8527	0.7654	0.8725 ± 0.0026	0.8548 ± 0.0089	0.9104 ± 0.0056
ABMIL	0.8492	0.8217	0.7416	0.8695 ± 0.0049	0.8643 ± 0.0121	0.9173 ± 0.0069
CLAM	0.8612	0.8372	0.7342	0.8657 ± 0.0071	0.8638 ± 0.0143	0.9127 ± 0.0100
TransMIL	0.9224	0.8527	0.8417	0.8752 ± 0.0083	0.8614 ± 0.0100	0.7933 ± 0.0172
CoOp	0.9133	0.8450	0.8401	0.8702 ± 0.0089	0.8667 ± 0.0035	0.9183 ± 0.0118
FiVE	0.9133	0.8760	0.8557	0.8771 ± 0.0095	0.8633 ± 0.0083	0.9183 ± 0.0035
baseline	0.9033	0.8527	0.8333	0.8695 ± 0.0031	0.8531 ± 0.0053	0.9046 ± 0.0035
**ConMIL**	**0.9324**	**0.8915**	**0.8600**	**0.8781 ± 0.0060**	**0.8667 ± 0.0089**	**0.9188 ± 0.0052**

baseline denotes the ConMIL backbone using PLIP-derived image features without text-guided semantic guidance.

Supplementary Table S3. Trainable parameter counts of the MLP and KAN classifiers.

Classifier	Trainable parameters
MLP	33,154
KAN	330,496

Supplementary Table S4. Learned semantic guidance coefficient α.

Dataset	α
CAMELYON16	0.5383
TCGA-BRCA	0.4937 ± 0.0293
